# Changes in thermal nociceptive responses in dairy cows following experimentally induced *Escherichia coli *mastitis

**DOI:** 10.1186/1751-0147-53-32

**Published:** 2011-05-18

**Authors:** Ditte B Rasmussen, Katrine Fogsgaard, Christine M Røntved, Ilka C Klaas, Mette S Herskin

**Affiliations:** 1 Trekantens Dyrlæger, Teglværksvej 42, DK-7000 Fredericia, Denmark; 2 Department of Animal Health and Bioscience, Faculty of Agricultural Sciences, Aarhus University, PO Box 50 DK-8830 Tjele, Denmark; 3 Department of Large Animal Sciences, Faculty of Life Sciences, University of Copenhagen, Bülowsvej 17, DK-1870 Frederiksberg C, Denmark

## Abstract

**Background:**

Mastitis is a high incidence disease in dairy cows. The acute stage is considered painful and inflammation can lead to hyperalgesia and thereby contribute to decreased welfare. The aim of this study was to examine changes in nociceptive responses toward cutaneous nociceptive laser stimulation (NLS) in dairy cows with experimentally induced *Escherichia coli *mastitis, and correlate behavioral changes in nociceptive responses to clinical and paraclinical variables.

**Methods:**

Seven Danish Holstein-Friesian cows were kept in tie-stalls, where the *E. coli *associated mastitis was induced and laser stimulations were conducted. Measurements of rectal temperature, somatic cell counts, white blood cell counts and *E. coli *counts were conducted. Furthermore, scores were given for anorexia, local udder inflammation and milk appearance to quantify the local and systemic disease response. In order to quantify the nociceptive threshold, behavioral responses toward cutaneous NLS applied to six skin areas at the tarsus/metatarsus and udder hind quarters were registered at evening milking on day 0 (control) and days 1, 2, 3, 6 and 10 after experimental induction of mastitis.

**Results:**

All clinical and paraclinical variables were affected by the induced mastitis. All cows were clinically ill on days 1 and 2. The cows responded behaviorally toward the NLS. For hind leg stimulation, the proportion of cows responding by stepping was higher on day 0 than days 3 and 6, and the frequency of leg movements after laser stimulation tended to decrease on day 1 compared to the other days. After udder stimulation, the proportion of cows responding by stepping was higher on day 1 than on all other days of testing. Significant correlations between the clinical and paraclinical variables of disease and the behavioral responses toward nociceptive stimulation were found.

**Conclusions:**

Changes in behavioral responses coincide with peaks in local and systemic signs of E. coli mastitis. During the acute stage of E. coli mastitis nociceptive thermal stimulation on hind leg and mammary glands results in decreased behavioral responses toward nociceptive stimulation, which might be interpreted as hypoalgesia.

## Background

Mastitis is a frequent production-associated disease in dairy cows, and is considered painful in the acute stage [1-4]. The severity of mastitis depends on the pathogen, host and environmental factors [5-7]. *Escherichia coli *provoke acute clinical mastitis characterized by marked increase in local inflammatory mediators accompanied by a strong systemic acute phase response. Cows are more sensitive to bacterial infection in early lactation, where local and systemic inflammatory signs are stronger than in mid or late lactation [7,8].

To date, bovine nociceptive responses have been quantified using mechanical [2,9] or thermal [10-12] stimulation of a hind leg. However, only few experiments have investigated the relationship between bovine mastitis and nociceptive responses [2,13] and none of them have used nociceptive stimulation directed at the udder. A Scottish field study involving cows with mild to moderate spontaneous mastitis with local but no systemic signs, found long term decreased nociceptive threshold, measured by mechanical cutaneous nociceptive stimulation on hind legs [2]. In contrast, Herskin *et al*. [13] found signs of increased nociceptive threshold in dairy cows with acute experimental *E. coli *mastitis and associated systemic symptoms when using thermal nociceptive laser stimulation (NLS) on hind legs. Whether these deviating results are due to the chosen stimulus modalities, type of bacteria, disease stage or disease severity is unknown.

The aim of the present study was to quantify changes in behavioral responses toward nociceptive stimulation in dairy cows in early lactation over a period of 10 days during and after experimentally induced *E. coli *mastitis.

## Methods

### Animals and housing

Eight Danish Holstein-Friesian cows, all in first lactation (30.9 ± 5.8 days postpartum), were housed in straw-bedded tie stalls (120 × 120 cm) with neck-bar ties of approximately 75 cm in the dairy barn facilities of Research Center Foulum, Aarhus University, Denmark. All cows were kept with empty neighbor stalls. The cows were fed twice daily at 8:00 h and 15:00 h with a total mixed ration (TMR) based on maize silage plus vitamins and minerals. Sufficient food was given to allow *ad libitum *intake. Cows had free access to water and were milked twice daily at 6:00 h and 17:00 h. Prior to experimental infection, the udder health as well as the general health were evaluated by clinical examination including measurement of rectal temperature, somatic cell count (SCC) and bacteriological examinations performed on milk samples, as well as white blood cell count (WBC) and glutaraldehyde test (Glutavac Test, Jørgen Kruuse A/S, Marslev, Denmark) performed on blood samples. Only cows free from major mastitis pathogens (i.e. gram negative bacteria, *Streptococcus agalactiae, Staphylococcus aureus, Strep. uberis, Strep. dysgalactia*), with a SCC < 100.000/ml milk, rectal temperature < 38.9°C, WBC < 10 × 106 cells/ml blood, and a negative glutaraldehyde test were included in the study.

Samples of liver [14] and udder tissue from the E. coli infected quarter and the matching control quarter [15] were collected from half of the animals as part of another experiment at 13 h and 24 h post inoculation. The animals received sedative and local anesthesia in relation to biopsy sampling [14,15].

The herd at Aarhus University is free of Infectious Bovine Rhinothracheitis, Bovine Virus Diarrhoea virus, Salmonella Dublin, and Strep. agalactiae and a number of severe cattle diseases according to the national disease status. In addition, herd screening for paratuberculosis indicated a low infection level.

All procedures involving animals were approved by the Danish Animal Experiments Inspectorate and complied with the Danish Ministry of Justice's law concerning animal experimentation and care of experimental animals. Members of the Danish Animal Experiment committee carried out inspection during the acute stage of the disease (J.no. 2006561-1197). This study followed a general treatment strategy allowing fluid therapy, non-steroid anti-inflammatory drugs and antibiotics to cows with severe clinical signs indicating shock. However, none of the cows received any medical treatment.

### Experimental design

**Figure 1:**
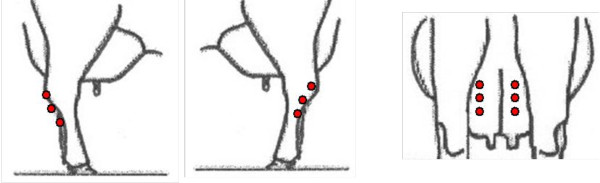
**Laser simulation aimed at udder and hind legs**. Graphic presentation of the position of the six single laser stimulations applied to the caudal part of the metatarsus on the hind legs and to the caudal part of the udder of dairy cows.

The study was designed as a longitudinal cohort study with the individual dairy cow being its own control. The nociceptive threshold, measured as behavioral responses, was measured once daily on day 0 (control day) and days 1, 2, 3, 6 and 10, by quantifying the animals' responses toward NLS (adapted from [12]) directed at the caudal part of metatarsus and udder (Figure 1). One day prior to inoculation, hair was trimmed from the tarsal joint to the pastern joint in order to standardize hair length (leaving approximately 0.5 cm). Udder hair was not trimmed, as the udders were trimmed regularly in the herd. On each day of testing, the hind legs and udder were brushed in order to remove manure, after which the cows were allowed a two minute adaptation period before start of laser stimulation. If a cow was lying down, she was forced to get up before brushing. On each test day, the computerized laser was placed on a trolley approximately two meters behind the cow to be tested. For half of the cows, stimulation was initiated on the udder, and the other half on metatarsus. Each test of nociceptive threshold - either at hind legs or udder - consisted of six successive laser stimulations; three on each hind leg or three on the left and right side of the udder, in a balanced order. In case of no responses toward laser stimulation, the maximum duration of laser exposure was 25 sec. Otherwise, the laser was turned off as soon as the cow responded behaviorally with one of the behaviors described in Table 1. If the cow started urinating, defecating, or performing other spontaneous movements, not directly caused by laser stimulation, the laser was turned off and the stimulation repeated. Between single simulations, behavior was observed during a 30 sec resting period (Table 1). For the individual cows, the same testing was repeated on each experimental day, where testing started at 13:00 h and continued for approximately two hours until all cows were tested. The two observers collected behavioral data by direct observation, entered into an on-site laptop, using special-made PC-software written for this purpose for keyboard operation.

**Table 1:** Ethogram of dairy cow behavior recorded during the tests of nociceptive responses

Behavioral variable	Definition
**During laser stimulation**	
Tail flick	The tail is flicked at least 5 cm to either side. A new event is recorded after a complete cycle of tail movement [12].
Tail pressing	The cow is pressing the central part of the tail against the base of the udder [13].
Muscle twitch	Contraction of single muscle group. A new event is recorded after a pause of at least 5 seconds.

**Type of initial response toward stimulation**	
Kicking	The hoof is thrusted against the floor or withdrawn at high speed [12].
Lifting leg	The hoof is lifted from the floor in a calm manner [12].
Stepping	The leg is moved, but the hoof is not lifted from the floor.
No response	The leg is not moved within the temporal limits of the test [12].

**Behavior during 30 s after exposure**	
Moving hind leg	At least one hind leg is moved. The hoof does not have to be lifted from the floor.
Licking leg	The cow licks the exposed hind leg or attempts to do so by turning the head against the hind leg.
Licking body	The cow licks other body parts than the hind legs.
Lying down	The cow changes posture from standing to lying.

### Induction of *E. coli *mastitis

A non-hemolytic *E. coli *Danish field isolate of (k2bh2) originally isolated from a cow with severe acute mastitis was used. All procedures involving handling of the inoculums were conducted in a laminar airflow bench under sterile conditions according to [15]. Each cow was inoculated with 10 ml of 0.9% pyrogen-free NaCl solution containing ~ 20-40 colony forming units of *E. coli *in the left front quarter immediately after evening milking at day 0. The right front quarter was treated as control and therefore not inoculated. Each teat was disinfected twice with cotton wool pre-wetted with 70% ethanol. The *E. coli*-NaCl solution was infused into the gland with a sterile teat cannula and the quarter was thoroughly massaged. After the inoculation the remaining bacteria suspension was retested in the laboratory for number of *E. coli *using large agar plates with Tryptone soy agar and Mac Conkey agar for 1 ml volume testing.

### Laser equipment

An adjustable 10 Watt (W) computer-controlled CO_2_-laser with a beam diameter of 0.6 cm and wavelength of 10.6 μm (Model 48-1, Synrad, Mukilteo, WA, USA) was used as the heat source. Attached to the CO_2_-laser was a visible cold He-laser pointer (Bantex, Denmark), which was used as aiming beam. The distance between the two laser beams was 4.5 cm. The applied laser intensities were 1.1 W and 1.8 W on the caudal udder and metatarsus, respectively.

### Clinical examinations and sampling

Clinical and paraclinical examinations were conducted daily. Anorexia was scored on a scale ranging from 1 to 4, with 1 being normal eating of TMR and 4 no eating observed. The udder was scored on a 1 to 4-scale with 1 being normal and 4 if at least one whole gland was warm, swollen, sore, firm and reddish. Milk appearance was scored daily on a 4-point scale with 1 being normal white homogenous milk and 4 being yellowish, serous milk with pus. Within one hour prior to the test of nociceptive responses, rectal temperature and K_3_EDTA stabilized blood samples were drawn from a sterile catheter in the jugular vein (inserted on d -1) and analyzed daily for WBC (106 cells/ml blood) on an automated hemocytometer (Cell-Dyn 3500, Abbot Laboratories A/S, Denmark). SCC was measured at milking using a DeLaval Cell Counter (DeLaval, Tumba, Sweden. Range 1-6000 × 10^3 ^cells/ml). To quantify *E. coli *(CFU/ml) and to rule out the presence of other mastitis causing pathogens, 10 ml foremilk were aseptically collected from the *E. coli *inoculated quarter as previously described [15].

### Statistical analysis

One cow did not test positive for E. coli and was excluded from the study. Due to technical difficulties, data from the udder stimulations and the SCC on d 2 contained only six cows. The final data set included observations from 83 tests of nociceptive responses consisting of a total of 498 successful laser simulations from 7 cows.

The behavioral variables were calculated for each cow, stimulation site (udder/hind leg) and day. During laser stimulation, the frequencies of tail flick and tail pressing were calculated per 25 sec. Furthermore, the median latency from onset of laser stimulation to first movement of hind legs was calculated, as well as the proportion of the type of movement - kicking, lifting leg, stepping or no response. Muscle twitch and lying down had very low representation in the data set and were excluded from further analyses. During the 180 sec observation period between the six laser stimulations, the following frequencies were calculated: Frequency of moving leg, licking body and licking leg.

Initially, the behavioral responses toward laser stimulation were analyzed separately for each body side (non-infected vs. infected) and compared using One Way Repeated Measures ANOVA (SigmaStat 3.1; Jandel Inc., San Jose, California). No significant differences were found, and data were pooled for the following comparisons. Subsequently, the behavioral responses to stimulation directed at the hind legs and the caudal udder on Day 0 vs. experimental days 1-10 were compared, using One Way Repeated Measures ANOVA (SigmaStat 3.1) when data followed a normal distribution. In non-normally distributed data the Friedman Repeated Measures ANOVA test on Ranks (SigmaStat 3.1) was used. When an effect of day was found, effect of biopsy status (biopsy vs. no biopsy) was investigated using a Two Way Repeated Measures ANOVA (SigmaStat 3.1) with day and biopsy status included as explanatory variables if P < 0.05. Biopsy status did not affect any behavior significantly and was therefore excluded from all statistical models.

Latencies to move the leg after initiation of laser stimulation were compared using survival analysis for right-censored data [16] and the PROC LIFETEST in SAS 9.1 (SAS Inst. Inc., Cary, NC, USA). For statistical day to day comparison of E. coli WBC, SCC, rectal temperature, and scoring of anorexia, milk and udder appearance, One Way Repeated Measures ANOVA (SigmaStat 3.1) was used for normally distributed variables and Friedman Repeated Measures ANOVA on Ranks in cases of lack of normality (SigmaStat 3.1).

PROC Spearman of SAS (SAS Version 9.1) was used to correlate behavior with SCC, rectal temperature, WBC and scores of anorexia, milk and udder appearance on day 1 and 2. Data are presented as mean ± SEM, except for the latencies, which are presented as medians followed by percentiles. In all analyses P < 0.05 was considered significant.

## Results

**Figure 2:**
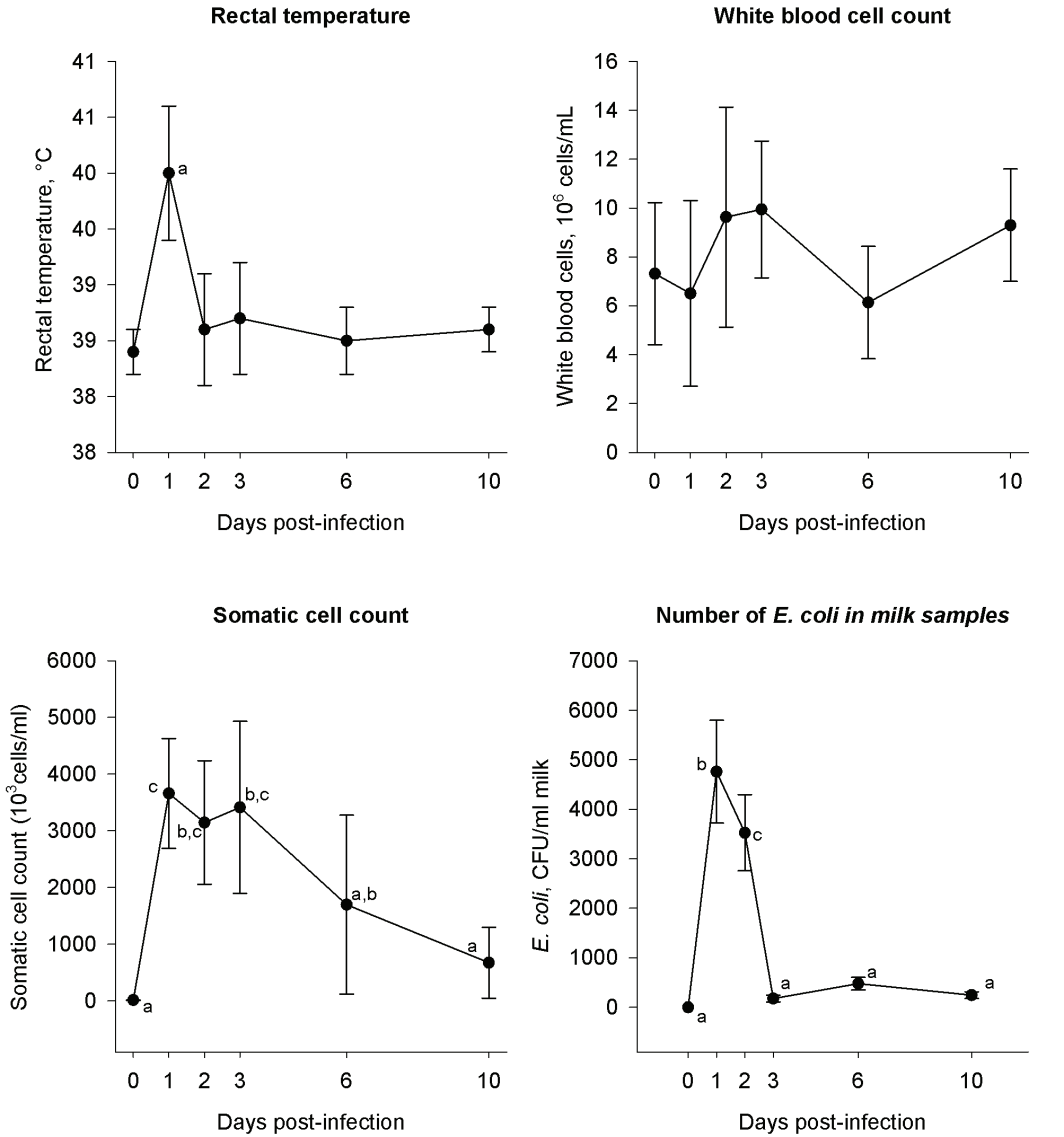
**The development of rectal temperature, white blood cells, somatic cell count and number of *E. coli *bacteria**. From one day before (day 0) until 10 days after inoculation with *Escherichia coli *into the udder of 7 dairy cows. Plots with different letters differ significantly. Error bars show SE.

**Figure 3:**
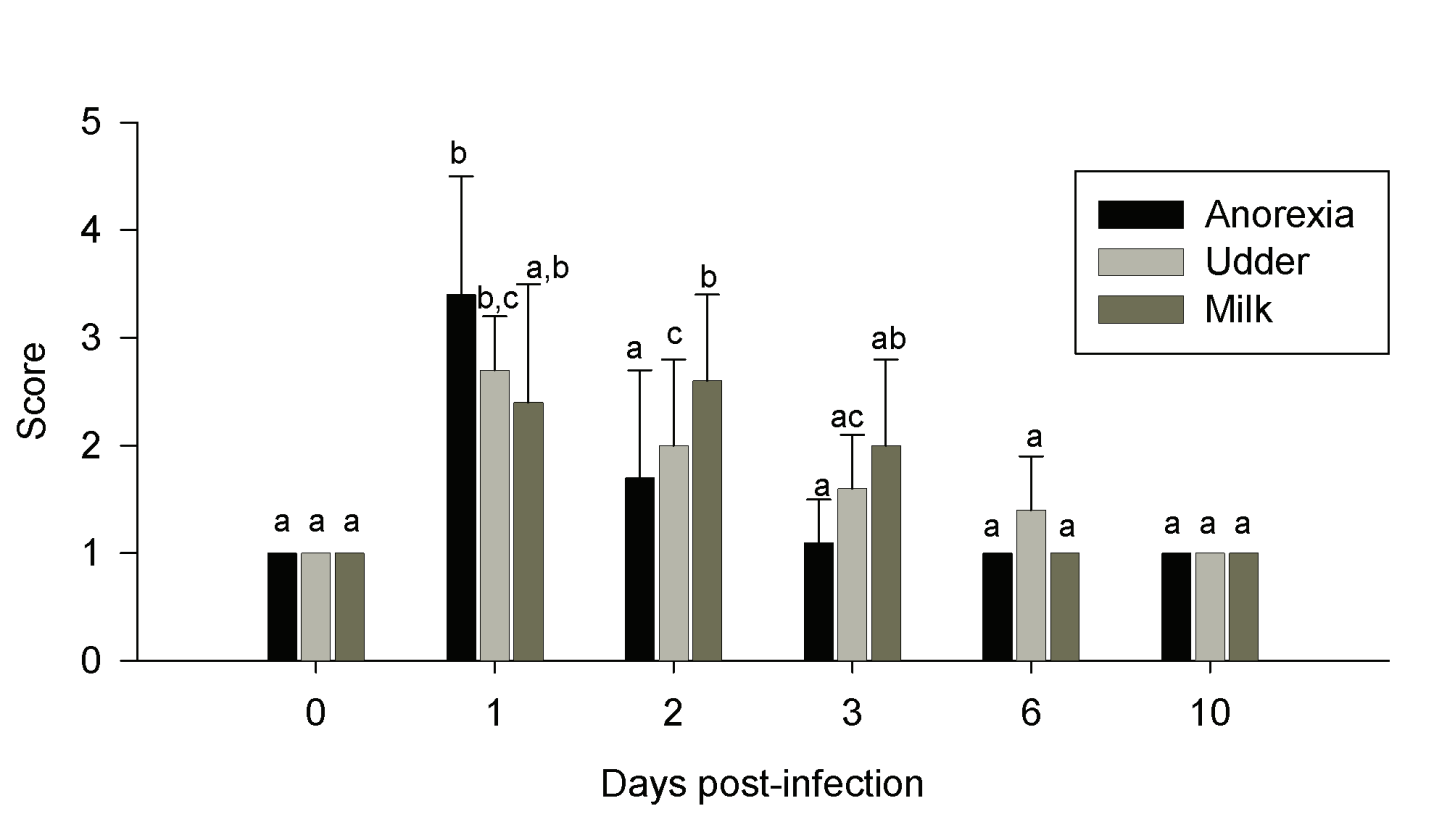
**Anorexia, udder and milk appearance**. Scaling of anorexia, udder and milk appearance from one day before (day 0) until 10 days after inoculation with *E. coli *into the udder of 7 dairy cows. Anorexia, udder and milk appearance were scaled ranging from 1 to 4 with 1 being normal and 4 highly affected. Plots with different letters differ significantly. Error bars show SE.

Bacteriological and clinical examinations confirmed *E. coli *mastitis in 7 cows (Figure 2 and 3). Rectal temperature (F_5,41 _= 14.7, *P *< 0.001), SCC (F_5,40 _= 12.2, *P *< 0.001), WBC (F_5,41 _= 6.1, P < 0.001), milk (χ^2 ^= 24.3 with 5 df, *P *< 0.001) and udder appearance (F_5,41 _= 14.4, *P *< 0.001), anorexia (χ^2 ^= 22.8 with 5 df, *P *< 0.001) and *E. coli *count (χ^2 ^= 29.2 with 5 df, *P *< 0.001) all changed after inoculation of *E. coli*.

### Nociceptive laser stimulation at hind legs

**Figure 4:**
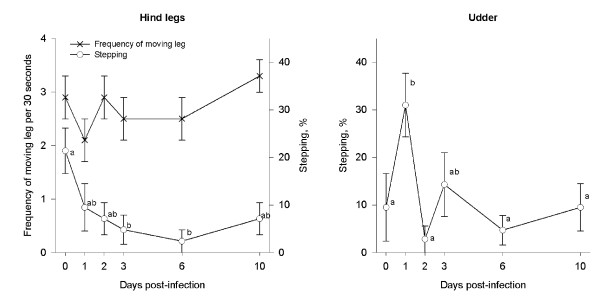
**Behavioral responses toward nociceptive laser stimulation**. Effects of *Escherichia coli *inoculation in the left front quarter on behavioral responses toward nociceptive laser stimulation at caudal udder or hind leg of dairy cows. Induction of *E. coli *mastitis was done after measurements on day 0, why day 0 serves as control day. Results are presented as mean and SE from 7 cows. Plots with different letters differ significantly.

For 5% of the single laser stimulations directed at the hind leg, no behavioral response was registered before the cut-off at 25 sec. The cows showed an overall median latency to move the leg of 5.5 sec (range 3-15) after stimulation, but the latency was not affected by the presence of mastitis (P > 0.1). The proportion of cows responding with the least forceful leg movement (stepping) differed between days (F5,41 = 3.24, P = 0.018) (Figure 4), and was numerically higher on day 0 than on all other days, as well as significantly higher on day 0 than on day 3 (t = 3.6, P = 0.004) and day 6 (t = 3.2, P = 0.003).

The proportion of cows responding with other types of leg movements did not differ between the experimental days. During laser stimulations, the cows responded with an overall mean of 6.3 ± 2.8 tail flicks and 0.1 ± 0.2 tail presses per 25 sec, but the frequencies of these behavioral events did not differ between the days of testing (P > 0.1). In the six 30 sec periods, immediately after each laser stimulation, a tendency was found for a changed frequency of leg movements between days (F5,41 = 2.2, P = 0.09) (Figure 4), with day 1 showing a tendency to decrease compared with the other days of testing (P < 0.1). Neither the frequency of licking leg (0.2 ± 0.2 per 30 sec) nor licking body (0.02 ± 0.02 per 30 sec) was affected by day of testing (P > 0.1).

### Nociceptive laser stimulation at the caudal udder

For 21% of all the simulations at the udder the temporal cut-off was reached. The overall median latency to move the hind leg was 13 sec (range: 4-25). However, the latency was not affected by the development of mastitis (P > 0.1). The proportion of cows responding to the laser by the least forceful leg movement (stepping) differed between days (χ^2 ^= 15.3 with 5 df, P = 0.009) and was significantly higher on day 1 than on all other days except day 3 (P < 0.004) (Figure 4). The proportion of cows responding with other types of leg movements did not differ between the experimental days. During laser stimulations, the dairy cows responded by an overall mean of 5.5 ± 2.5 tail flicks and 0.5 ± 1 tail presses per 25 sec, but the frequencies of these behavioral events did not differ during the study (P > 0.1). Neither of the behaviors measured in the 30 sec periods after each laser stimulation were affected by the presence of mastitis.

### Correlations between clinical/paraclinical and behavioral registrations

Out of the 40 possible correlations (20 on each of days 1 and 2), five turned out to be significant or to be tendencies. On day 2, the SCC correlated negatively with the proportion of leg lifting in response to laser stimulation directed at caudal udder (*P *= 0.005, r = -0.97), and tended to correlate positively with the proportion of kicking (*P *= 0.06, r = 0.87). On day 1, the rectal temperature correlated positively with the latency to move the leg after laser stimulation directed at the caudal udder (*P *= 0.02, r = 0.84). Furthermore, on day 1, WBC correlated negatively with the latency to move the leg during laser stimulation directed at the hind legs (*P *= 0.0008, r = -0.95). Finally, on day 2, the anorexia score correlated negatively with the latency to move the leg after laser stimulation directed at the hind legs (*P *= 0.03, r = -0.85).

## Discussion

This is the first report to present behavioral responses of mastitic dairy cows toward NLS directed at hind legs and caudal udder. The study shows that the cows responded behaviorally toward NLS directed at the udder, as shown by avoidance movements of the hind legs. One day after the inoculation of E. coli into the mammary gland, the cows developed acute local and systemic clinical signs of mastitis, classified as mild to moderate [8,17]. At this time, an increased proportion of behavioral responses with the least forceful leg movement - stepping - was observed. Furthermore, leg movements during the 30 sec period after the NLS tended to be decreasing. These changes suggest that the cows experienced hypoalgesia associated with the acute clinical mastitis. The findings are quite unexpected as the release of inflammatory mediators in the udder at a high level is expected to increase the risk of hyperalgesia [18]. Furthermore, the results are in contradiction with findings by Fitzpatrick et al. [2] who showed evidence of a period of hyperalgesia in dairy cows after spontaneous mastitis. However, the present data confirm our previous findings of increased nociceptive threshold in mastitic dairy cows [13]. Unfortunately, neither the present study nor our previous study [13] included stimulation with a non-painful stimulus thus limiting the possibility to conclude whether the decreased responses were due to hypoalgesia or a generalized decreased reactivity.

In a recent trial, reduced self-grooming and feeding activity were observed during the acute stage of induced bovine E. coli mastitis [19]. Hence, it is possible that the increased nociceptive threshold on day 1 was due to a disease-induced decrease in general responsiveness [20] and not hypoalgesia. The present correlations between behavioral measurements of nociceptive threshold and the clinical/paraclinical signs of mastitis on day 1 may support this suggestion, as they express the severity of the disease. However, more research is needed in order to clarify this.

In the present study, differences between behavioral responses toward NLS directed at the infected vs. the non-infected body side was not found. Contrarily, Kemp et al. [9] found that mastitic cows have a higher mechanical threshold on the non-infected as compared with the infected side, whereas Fitzpatrick et al. [2] observed the lower threshold on the infected side in cows with spontaneous mastitis without systemic reactions. Studying the mammary secretion of the inflammatory peptide bradykinin during mastitis, Eshraghi et al. [3] showed that the mammary release of the inflammatory mediator did not only occur in the infected quarter, but also in the other mammary glands. During acute E. coli mastitis, an inflammatory response is present both in the infected as well as the non-infected quarters, however, with marked local inflammation only in the infected quarter [21]. Based on these findings, it is possible that nociceptive threshold can be affected on both sides. A possible explanation to the earlier results showing differences between sides could be that the cows, e.g. due to soreness in the affected gland, change their inclination to move the leg on that side. However, with only seven cows in our study, minor differences between body sides may have gone undetected due to the limited statistical power.

Control stimulations at the two sites performed on day 0 triggered a comparable frequency of tail flicks, while the occurrence of tail pressing was only observed after stimulation of the udder. The latter behavior has been suggested to be a sign of pain during milking of mastitic cows [13], and the present results might support this, even though proper validation of behavioral responses to udder pain has not taken place yet.

The applied intensities of the laser beam of 1.1 W for udder stimulation and 1.8 W for hind leg stimulation were based on data from Herskin et al. [12]. In the present study, the protocol used for udder stimulation was adjusted before the experiment by a small pilot study including five non-experimental cows. Here, it was shown that udder stimulation using 1.1 W triggered a behavioral response with a latency of approximately 10 sec, whereby the experimental cows would be able to show bi-directional changes in nociceptive threshold within the present test. The observed overall median latency to leg movement after udder stimulation was numerically higher than the latency after stimulation directed at the hind legs and more cows did not respond within the cut-off period when stimulated at the udder. However, the present results suggest that the selected power output of 1.1 W for udder stimulation was sufficient to trigger behavioral responses from the majority of the cows.

As no control group was available for the present study, all seven cows were tested on day 0 and these data were treated as control observations. One drawback of such a design may be that the cows learn to associate the presence of observers and test equipment in the barn with the expectation of an aversive stimulus, and thus become able to respond earlier and perhaps stronger after repeated testing [22]. To avoid sensitization of the skin and peripheral nociceptors a 24 h interval between tests was chosen, as healthy dairy cows do not show significant changes in behavioral responses toward NLS temporally separated by 24 h [23]. Similarly, neither Rushen et al. [24] using dairy cows, nor Veissier et al. [11] examining nociceptive threshold in healthy Holstein bull calves found effects of repeated testing with an interval of 24 h. In the present study, increased responding with the least forceful behavioral response on day 0 as compared to days 3 or 6 might indicate, that the cows were responding stronger later in the study either due to associative learning, sensitization or hyperalgesia due to the mammary gland inflammation. However, clarification of this warrants further study.

## Conclusion

Changes in behavioral responses toward NLS directed at hind legs and caudal udder of mastitic dairy cows coincided with peaks in local and systemic signs of *E. coli *mastitis. During the acute stage of *E. coli *mastitis, NLS on hind legs and mammary glands led to decreased behavioral responses, which may be interpreted as hypoalgesia.

## Competing interests

The authors declare that they have no competing interests.

## Authors' contributions

DBR participated in the design of the study, carried out the pilot study and the tests of pain sensitivity, the clinical registrations as well as drafted the first version of the manuscript. MSH enabled lending of the laser equipment, participated in the design of the study, pilot study and performed the statistical analysis. KF has drafted major parts of final manuscript, the graphical figures and contributed to the statistical analysis. CMR was responsible for the experimental induction of mastitis, paraclinical measurements and for the overall experimental plan. ICK contributed to draft the manuscript. All authors read and approved the final manuscript.

## References

[B1] Milne MH Nolan AM Cripps PJ Fitzpatrick JL Assessment and alleviation of pain in dairy cows with clinical mastitis Cattle Pract 2003 11 289 293

[B2] Fitzpatrick JL Young FJ Eckersall D Louge DN Knight CJ Nolan A Recognising and controlling pain and inflammation in mastitis Proc of the British Mastitis Conference 1998 36 44

[B3] Eshraghi HR Zeitlin IJ Fitzpatrick JL Ternent H Logue D The release of bradykinin in bovine mastitis Life Sci 1999 64 1675 1687 10.1016/S0024-3205(99)00105-8 10328527

[B4] Leslie K Kielland C Millman S Is mastitis painful and is therapy for pain beneficial? NMC Annual Meeting Proceedings 2010 Wi, USA

[B5] Barkema HW Schukken YH Zadoks RN Invited review: The role of cow, pathogen, and treatment regimen in the therapeutic success of bovine *Staphylococcus aureus *mastitis J Dairy Sci 2006 89 1877 1895 10.3168/jds.S0022-0302(06)72256-1 16702252

[B6] Pyorala S Mastitis in post-partum dairy cows Reprod Dom Anim 2008 43 Suppl 2 252 259 10.1111/j.1439-0531.2008.01170.x 18638132

[B7] Fox LK Prevalence, incidence and risk factors of heifer mastitis Vet Microbiol 2009 134 82 88 10.1016/j.vetmic.2008.09.005 18930610

[B8] Burvenich C Van Merris V Mehrzad J Diez-Fraile A Duchateau L Severity of *E. coli *mastitis is mainly determined by cow factors Vet Res 2003 34 521 564 10.1051/vetres:2003023 14556694

[B9] Kemp MH Nolan AM Cripps PJ Fitzpatrick JL Animal-based measurements of the severity of mastitis in dairy cows Vet Rec 2008 163 175 179 10.1136/vr.163.6.175 18689778

[B10] SchwartzkopfGenswein KS Stookey JM dePassille AM Rushen J Comparison of hot-iron and freeze branding on cortisol levels and pain sensitivity in beef cattle Can J Anim Sci 1997 77 369 374 10.4141/A96-127

[B11] Veissier I Rushen J Colwell D de Passille AM A laser-based method for measuring thermal nociception of cattle App Anim Behav Sci 2000 66 289 304 10.1016/S0168-1591(99)00099-4 10700628

[B12] Herskin MS Müller R Schrader L Ladewig J A laser-based method to measure thermal nociception in dairy cows: Short-term repeatability and effects of power output and skin condition J Anim Sci 2003 81 945 954 12723084 10.2527/2003.814945x

[B13] Herskin MS Røntved CM Nielsen L Nielsen NI Behavioural and nociceptive changes during milking of dairy cows with an experimentally induced *E. coli *mastitis Proc of the 4th IDF International Mastitis Conference 2005 940

[B14] Vels L Rontved CM Bjerring M Ingvartsen KL Cytokine and acute phase protein gene expression in repeated liver biopsies of dairy cows with a lipopolysaccharide-induced mastitis J Dairy Sci 2009 92 922 934 10.3168/jds.2008-1209 19233785

[B15] Buitenhuis B Edwards SM Røntved CM Ingvartsen CL Sørensen P Global gene expression of the mammary gland in Holstein cows with *Escherichia coli *induced mastitis BMC Genomics 12 130 10.1186/1471-2164-12-130 PMC3053262 21352611

[B16] Kleinbaum DG Statistics in the health science. Survival analysis - a self-learning text 1996 Springer-Verlag New York, Inc

[B17] Hirvonen J Eklund K Teppo AM Huszenicza G Kulcsar M Saloniemi H Acute phase response in dairy cows with experimentally induced *Escherichia coli *mastitis Acta Vet Scand 1999 40 35 46 10418194 10.1186/BF03547039 PMC8043231

[B18] Flecknell P Waterman-Pearson A WB Saunders Pain Management in Farm Animals 2000 1

[B19] Fogsgaard K Effects of disease on behaviour and management of dairy cows 2010 Aarhus University, Denmark M.Sc. Thesis

[B20] Hart BL Biological basis of the behavior of sick animals Neurosci Biobehav Rev 1988 12 123 137 10.1016/S0149-7634(88)80004-6 3050629

[B21] Mitterhuemer S Petzl W Krebs S Mehne D Klanner A Wolf E *Escherichia coli *infection induces distinct local and systemic transcriptome responses in the mammary gland Reprod Dom Anim 2010 45 34 35 10.1186/1471-2164-11-138 PMC2846913 20184744

[B22] McFarland D The Oxford Companion to Animal Behaviour 1981 1 Oxford University Press

[B23] Herskin MS Munksgaard L Ladewig J Effects of acute stressors on nociception, adrenocortical responses and behavior of dairy cows Physiol Behav 2004 83 411 420 10.1016/j.physbeh.2004.08.027 15581663

[B24] Rushen J Boissy A Terlouw EMC de Passille AMB Opioid peptides and behavioral and physiological responses of dairy cows to social isolation in unfamiliar surroundings J Anim Sci 1999 77 2918 2924 10568459 10.2527/1999.77112918x

